# High-throughput isolation of ultra-pure plasmid DNA by a robotic system

**DOI:** 10.1186/1472-6750-6-9

**Published:** 2006-02-16

**Authors:** Volker Kachel, Georg Sindelar, Stefan Grimm

**Affiliations:** 1 Max-Planck-Institute for Biochemistry, Am Klopferspitz 18a, 82152 Martinsried, Germany; 2 Imperial College London, Du Cane Road, London W12 0NN, UK

## Abstract

**Background:**

With the availability of complete genomes, a systematic inventory of cellular processes becomes achievable. This requires assessing the function of all individual genes. Transfection of plasmid DNA into cell culture cells is an essential technique for this aim as it allows functional overexpression or downregulation of genes. While many robotic systems isolate plasmids for sequencing purposes, for more demanding applications such as transfections there is a shortage of robots for the high-throughput isolation of plasmid DNA.

**Results:**

Here we describe a custom-made, automated device, which uses a special protocol to isolate plasmid DNAs with a purity sufficient for efficient transfections into mammalian cells. Approximately 1,600 ultra pure plasmids can be isolated in a 96-well plate format within 12 hours. As a unique feature the robot comprises the integration of a centrifuge instead of expensive columns, the use of a custom-made pipetting head with a movable gripper, especially designed shaking platforms and an acetone wash facility.

**Conclusion:**

Using this robot we demonstrate how centrifugation steps with multiple precipitations, most notably through a precipitation step of SDS in isopropanol, lead to high purity plasmid DNA and make possible high-throughput transfections into mammalian cells for functional gene annotations.

## Background

After the completion of the sequence from several genomes including the human the novel field of systems biology is seeking a thorough functional understanding of all cellular components [1,2]. To this end it is necessary to assign functions to an unprecedented number of genes. The recent finding that the human genome comprises only about 25,000 genes appears to make a complete functional inventory easily feasible [3]. However, it is estimated that the number of splice isoforms greatly exceeds this figure and that most genes harbour multiple functional activities. In addition, the recent introduction of RNAi libraries facilitates knock-down experiments in cell culture but the active RNAi sequence cannot be inferred from the sequence alone making multiple constructs necessary [4,5]. In addition, functional testing of cis-regulatory DNA sequences are envisaged in systems biology and require the construction of numerous reporter plasmids and their efficient introduction into cells [6]. Moreover, since many gene activities cannot be assayed in selections and testing gene pools is often not sensitive enough, functional high-throughput screens of individual genes are required. Consequently, to achieve these aims robotic plasmid DNA isolations are needed, which yield DNA with a purity sufficient for efficient transfections[7]. We have established an assay for dominant, apoptosis-inducing genes as an example of a function that can only be targeted by a screen in contrast to a selection [8]. This approach requires an individual DNA isolation, transfection and functional assay for every cDNA clone of a gene library [9]. A number of genes were already isolated by this approach and their analysis demonstrates that they mediate specific signals for apoptosis [8,10-13]. Recently, we introduced a transfection robot to increase the throughput in this screen [14]. However, a robot to isolate the plasmid DNA from the complex mixture of the bacterial lysates was still lacking. So far, robotic devices yield plasmid DNA that is only suitable for sequencing, which is much less demanding on the purity of the DNAs [15-18]. Here we reveal the construction and use of this custom-made robot for the isolation of ultra-pure plasmid DNA for efficient transfections that can be used for many additional functional high-throughput screens.

## Results and discussion

### Layout of the robot

**Figure 1:**
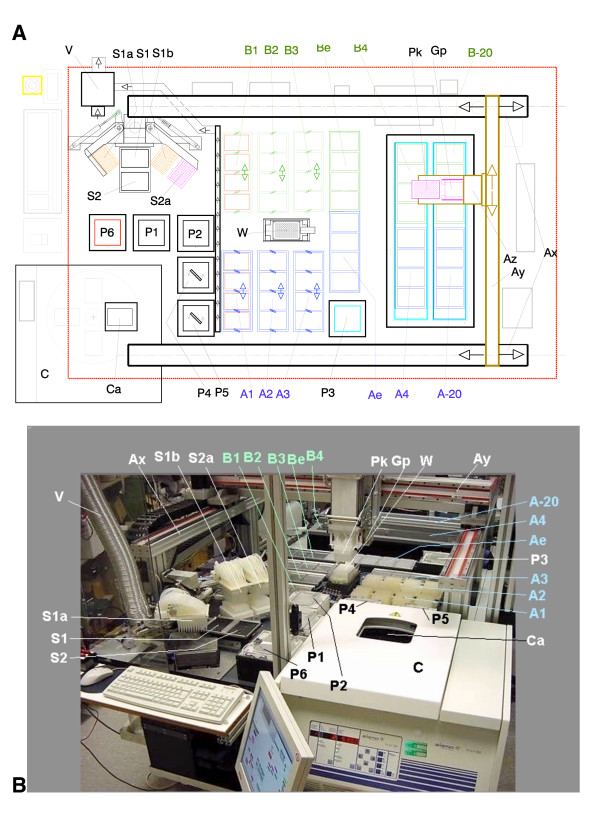
Layout of the DNA isolation robot (A) and photographic view of the machine (B). Ax: Rails for x-motion of the pipetting and gripper head; Ay: Rail for y-motion of the head; Az: Rail for z-motion of the head; A1, B1: Platforms with heater and shaker for 96 fold deep-well-plates (DWPs); A2, B2, A3, B3: Platforms with shaker for 96-fold DWPs; Ae, Be: Static platforms for 96-well microtiter-plates (MTP); A4, B4: thermo-isolated DWP-platforms cooled to 4°C; A-20, B-20: thermo-isolated DWP-platforms with lid cooled to -20°C; C: Centrifuge with 4-fold rotor; Ca: Loading position of the centrifuge; P1, P2: Reservoir of pipetting fluid (Room temp); P3: Reservoir of pipetting fluid (4°C); P4, P5: Reservoir of pipetting fluid with integrated stirrer (room temp); P6: Reservoir of pipetting fluid with heater up to 80°C; Pk: Movable 96-fold pipetting head; Gp: Gripper integrated into the movable head for transporting DWPs; S1: Platform for a 96-fold DWP for loading and withdrawing acetone; S1a: Tiltable 96-fold pipetting head for supplying acetone; S1b: Tiltable 96-fold pipetting head for withdrawing acetone; S2: Platform for a 96-fold DWP for withdrawing the bacteria medium; S2a: Tiltable 96-fold pipetting head for withdrawing fluids; V: Ventilation for removing acetone vapour; W: Parallel washer for the tips of the 96-fold pipetting head.

Figure 1 presents the layout of the robot platform, which contains an x-y-z-gantry system (dimensions: 160 × 90 × 25 cm). This makes the enclosed space accessible for both the movable pipetting head, which is equipped with standard 200 μl pipetting tips, and the attached gripper, which transports the 96-well deep-well plates (DWPs). A PC-controlled centrifuge (Sigma 4K15R) was incorporated, which is equipped with a sliding closing in its cover and allows the gripper to vertically insert and remove 96-well DWPs. The solutions P1 to P6 are kept in lidded containers, arranged around the centrifuge, which open only when the pipetting head arrives for fluid uptake. Opposite the centrifuge, we have located the S1/S2 pipetting station that comprises a sucking and a dispensing 96-well head for acetone and operates independently of the movable pipetting head to avoid contaminations with acetone. One additional platform and a separate pipetting head in this pipetting station are exclusively designated for the removal of bacteria supernatants. It also contains an integrated ventilation system that eliminates dangerous vapour concentrations of this ketone. Each robot run handles two sets of four DWPs in parallel as interlaced processes A and B. The platforms that accommodate these DWP groups are arranged side-by-side to allow rapid transport of supernatants between them. Some of the platforms such as A1,2,3 and B1,2,3 are equipped with shaking functions for mixing solutions or for re-suspending bacteria- and silica-pellets. Platforms A1 and B1 can be heated to evaporate acetone. Other platforms are inserted into isolated cooling boxes containing 4°C and -20°C compartments to facilitate incubations at these temperatures (A5, B5 and A6, B6). Refrigerating machines placed under the working platform pump cooling fluid through these cooling boxes. While the 4°C box is unlidded, the -20°C slot features a PC-controlled cover to reduce the generation of ice inside the box. A centrally located washing station for the movable pipetting head is used to clean the tips after each pipetting step. The interplay of the different robotic parts is directed by a PC. The various stations of the process were arranged on the platform so that minimal space is occupied. Consequently, the platforms were concentrated on one end and the fluid reservoirs grouped around the centrifuge, next to the pipetting station.

### Process overview

The robot was specifically tailored to the needs of our particular DNA isolation protocol, which uses alkaline lysis of bacteria and binding of plasmid DNA to silica oxide[19], but deviates from conventional protocols in that (i) it does not include chaotropic substances for the binding of DNA to silica oxide, (ii) it removes LPS (endotoxin) by co-precipitation with SDS in isopropanol and (iii) it eliminates further residual impurities by an acetone wash. Besides the integration of the computer-controlled centrifuge, a number of additional modifications had to be introduced compared with the original manual protocol to accommodate for the requirements of a robotic system [19]. The transfer of the supernatant, for example, which was formerly accomplished by tilting the plates, is now performed by the pipetting head. The mixing of solutions and the resuspension is done by dedicated mixing platforms instead of a vortex machine. For the drying of the silica pellets we switched from incubations in temperature-controlled incubators to heated platforms and blowing air from the 96-well pipettor. The acetone washing process is now performed by a designated set of pipettors. For cooled incubation, lidded containers were established on the platform, which substitute the refrigerator incubations in the previous protocol. During a single run, incubation- and centrifugation times sum up to about 90 minutes time of robot inactivity. This time is efficiently used by running a second, interlaced process. In this way, the overall processing time of one run is extended from 240 to 270 min, the throughput, however, is doubled to 768 samples. Prior to the run the platforms A3 and B3 are manually supplied with eight DWPs containing LB medium with recombinant bacteria. Platforms A1, B1 and A2, B2 are filled with empty DWPs into which the solutions with the plasmid DNAs at various stages of the purification process are transferred. The platforms Ae and Be contain empty 96-well microtitter plates (MTPs) to store the final DNA samples. For simplicity only the process of one group of plates is described here. The run starts with the transport of four DWPs from platform A3 to the centrifuge. After one carrier is loaded the centrifuge automatically turns the rotor to the next position and arrests it by the insertion of a bolt. The gripper can then deposit the next DWP at defined coordinates. After centrifugation the plates are transferred to the pipetting station S1/S2 and the supernatants are removed from the bacteria pellets by a designated pipetting head. The plates are moved to the platform A3 and solution P1 is added by the movable pipettor. The four DWPs are fixed on the platform, which is pneumatically set into linear vibration with pre-selected frequency and duration to resuspend the bacteria pellet. When the fixing bolts are released, the platform is set to its stop coordinates for access by the pipetting head. After addition of solution P2, gentle shaking and incubation for 5 min, P3 is added, the solutions are incubated at -20°C for 5 min, mixed again and after centrifugation the supernatant is pipetted to a new plate (A2). P4, which contains SDS in isopropanol, is added, mixed and the plates are incubated first at 4°C (15 minutes) on platform A4 and then at -20°C (15 minutes) on platform A-20. After another centrifugation step the supernatants are removed and added to new DWPs on platform A1. Silica oxide is dispensed, mixed and the interaction with the plasmid DNA facilitated by incubation at RT for 20 minutes. The plates are again centrifuged and washed twice with acetone with removal of the supernatant at each step. Drying the silica pellets by blowing air into the 96-well plates through the pipetting head, their re-suspension in water (150 μl) and the subsequent centrifugation yields the pure plasmid DNA in the supernatant, which is pipetted into MPTs on platform Ae.

### Process results

**Figure 2:**
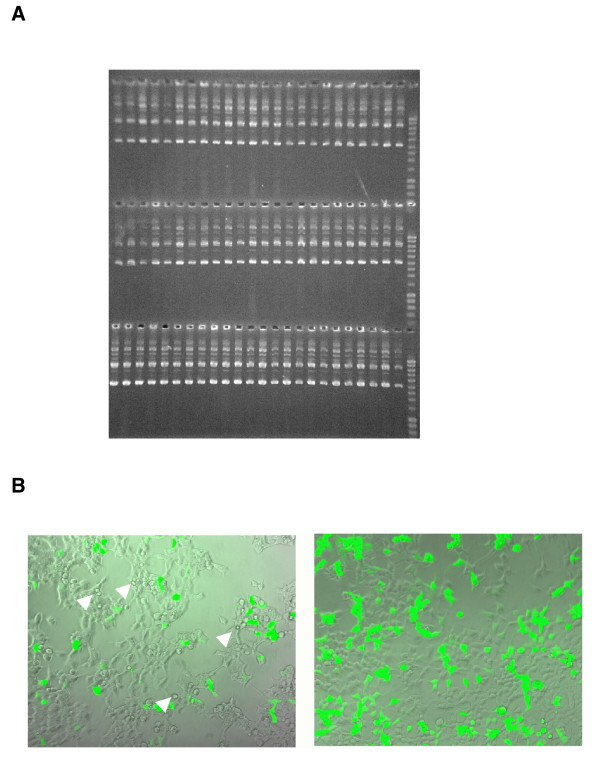
Plasmid yield of a robot run (A) and comparison of transfection efficiencies of plasmid DNAs isolated by the robot and a commercial kit (B). (A) pLantern plasmid DNA was extracted by the robotic system. Probes were chosen randomly from four different plates of processes A and B, 10 μl of plasmid solution per slot were applied to a 1% agarose gel and visualised by ethidium bromide staining. Marker lane: 4 μl Smart Ladder (Eurogentec). The highest band (10 kb) is 80 ng, the band at 3 kb corresponds to the lowest plasmid band and is 24 ng. (B) Transfection of column-isolated GFP plasmid DNA versus robot-isolated plasmid DNA. 293 cells were transfected with equal amounts of DNA in a 24-well format. After 30 hours phase contrast and fluorescent microphotographs were taken at a 100fold magnification with a Zeiss Axiovert S100TV equipped with a FITC-filter. Arrows denote damaged cells in the transfections.

One run takes 270 minutes and purifies 768 DNA samples from eight 96-well plates in two parallel processes. We tested the reproducibility of the DNA isolations with the robot and observed a uniform plasmid DNA yield (Fig. 2A) with 50% of the plasmids in the supercoiled form. The 260/280 OD ratio is around 1.8 in these preparations. Final plasmid concentrations are usually 100 ng/μl in 150 μl water. These parameters are routinely measured in selected samples after each run. Our calculations show that one DNA plasmid purification costs 5,5 cents/well which is much less than the costs of commercial columns. Aliquots were removed at different stages of the process and investigated for their plasmid DNA content. As expected, the biggest plasmid loss of about 50% occurs during the SDS precipitation in P4 (data not shown). To compare the quality of the plasmids we isolated pLantern, a GFP expression plasmid using in parallel our robotic system and commercially available 96-well silica columns. The GFP fluorescence and the state of the cells were assayed after transfection of equal amounts of plasmid into the cells. Figure 2B reveals that the DNAs from the commercial columns were less efficiently transfected and also adversely affected the cells as evident by their detachment and rounding up (arrows). The latter observation is of crucial importance, since, while transfections with less well purified plasmid DNA could potentially be obtained in some cells, the effects of the plasmid DNA can only be observed if the cells are not damaged by residual bacterial components[20]. We concluded that our robotic plasmid preparations are superior both in costs and quality compared to the currently available ways to obtain DNA in a 96-well format.

The DNA isolation robots presented here complements the transfection robot that we have described earlier[14]. Both together establish a high-throughput screen for the determination of individual gene activities. Irrespective of whether this more conventional way of introducing DNA into cells is used or recently described transfection alternatives such as reverse transfetions on microarrays, both require the purification of DNA in a high-throughput format and justify the set-up of such a DNA isolation robot [21]. We have already used this robot to screen for apoptosis inducing genes and isolated several genes that cause cell death.

Potential further developments of this robot will address the miniaturisation of the process. Also, magnetic beads could replace the use of the centrifuge, although our preliminary results implicate that commercially available beads still co-purify a considerable amount of contaminations (not shown) and consequently require improvements.

## Conclusion

A robot for the isolation of plasmid DNA with high purity has been developed. It is tailored to our protocol of plasmid isolation that uses centrifugations steps instead of expensive columns. Purification in this protocol is achieved by multiple precipitations, most notably though a precipitation step of SDS in isopropanol, which removes endotoxins from the bacterial lysates. Systematic functional gene annotations necessitate efficient high-throughput transfections into mammalian cells. This requires higher quality DNA than those obtained with robots currently used to isolate plasmid DNA for sequencing reactions. Thus, our robot extracts ultra-pure plasmid DNA from the complex mixture of bacterial lysates and makes possible functional high-throughput screens based on iterative cell transfections.

## Methods

The following section comprises a brief explanation of the technical features. Additional details can be obtained online in the supplementary information [see Additional file 1].

### The gantry system

A five axis-gantry robot with stepping motors (working field x = 160 cm y = 90 cm, z = 25 cm) moves the main pipetting head mounted on the z-sled (Z1, see supplementary information). The 96-tip pipettor is the same as the one used in our transfection robot [14]. It has access to all platform positions. Its movable rollers are controlled by the 4^th ^axis of the gantry system (Z2). The 5^th ^axis moves the S1/S2 pipettors up and down (Z3). The five axes are driven by the gantry robot controller, which is operated by ASCII strings from the PC via the serial1 interface. The resolution of the x- and y-axis is 10 steps/mm, that of the spindle-driven is z-axes 80 steps/mm. Maximum speed is 8000 steps/sec. A pneumatically operated gripper for the transport of the 96-well plates between the locations of the working platform is attached to the Z1-sled immediately behind the pipetting head. A two-step z-move is used for the operation of the gripper. The z1 sled moves the gripper to the z1 base position and a built-in pneumatic cylinder drives it further down a fixed distance of 20 cm to the DWP-gripping or -releasing position.

### Wash station for pipetting tips

The tips of the 96 main pipetting head are manually plugged onto Gilford type holder cones and arranged in an 8 × 12 matrix of 9 mm tip distance. The tips are cleaned in a tip washing station in-between pipetting tasks. The washing station consists of 96 vertical tubes through which cleaning water can be pumped upwards. For cleaning, the 96 tips of the head are inserted into the washing tubes. When the water pump is switched on, the up-flowing water cleans the outside of the tips. Simultaneously, cleaning fluid is sucked into the interior of the tips. The contaminated water overflowing at the top of the tubes is drained away into a waste container. The tips are moved out of the washing tubes and positioned over the gaps between the tubes where the contaminated fluid is blown out and drained away. Such cleaning cycles can be programmed as required, each lasting about 5 seconds. As fresh water is used only when the tips are inserted into the washing tubes, it ensures low consumption of cleaning water.

### PC-controlled centrifuge

A PC-controlled cooled centrifuge (Sigma 4K15 robotic; Sigma, Osterode, Germany) for the simultaneous centrifugation of four 96-well DWPs was incorporated into the robot. The centrifuge is controlled by ASCII-string commands via the serial 2 interface of the PC. Automatic loading and unloading is performed through a hatch in the centrifuge lid. At the start of the centrifugation process the centrifuge is checked for imbalance. If an imbalance has been detected the centrifuge stops, an alarm is triggered and the program execution is set to "wait". The system supervisor may then manually balance the DWPs and continue the process execution.

### Pipetting station S1/S2

The z-motion (Z3) of the three pipetting heads of the station S1/S2 is controlled by the gantry robot controller. They work in accordance with the "suction-pressure" principle [14]. Suction and pressure are generated in a shared air tube, which branches out into the 96 pipetting tips. This simple principle allows the dispensing and take-up of equal volumes of fluid and works well with tubes of identical length between the common tube and the pipetting tips. The gripper transport system transfers the DWPs to the pipetting station platforms S1 and S2. Platform S2 serves as suction station independent of the main pipetting head. It is used for removing the high volume of bacteria supernatant after the first centrifugation. On platform S1 acetone is dispensed and removed to wash the plasmid DNAs. The 96-fold pipetting head S1a and the joined heads S1b and S2a are swung out when a DWP is delivered by the gripper and placed on S1. The cover is opened, the cooled acetone vessel moves up and the pipetting head S1a is lowered until the tips are immersed into the acetone. By a short suction pulse about 200 μl acetone is taken up by the tips. The head is moved up, swung over the DWP, moved down until the tips enter the top of the wells and the acetone is blown out by a smooth pressure pulse. Cooling of the acetone is required in order to reduce the acetone vapour pressure. For acetone removal the DWP is also placed on S1, the head S1b is moved over the DWP, lowered down until the tips are immersed into the acetone supernatant and the supernatant is taken up by a short suction pulse. S1b is moved up, swung over the waste uptake S1w and the content of the pipetting tips is blown out. The number of suctions, the depth of immersion and the strength and duration of the suction pulses are experimentally determined. The complete acetone washing process consists of: Transport of the DWPs containing the silica pellets to the S1 platform (removal of acetone if acetone has been added in a previous step), addition of 400 μl acetone, transport to the A1/B1 platform, shaking and transport to the centrifuge for centrifugation.

### Fluid supply vessels

The six fluid supply vessels P1 – P6 consist of delrin plastic holders and polyetylene insert vessels, which can be filled, emptied and cleaned outside the robot. All vessels are covered by pneumatically operated lids. The functional space (FSP) below the insert vessels allows the insertion of additional elements like stirrers, coolers and heaters. The FSPs of P4 and P5 contain electronic stirrers. P3 is equipped with a cooling element that is supported by the 4°C cooler. P6 contains an electrically driven 60°C heater. The FSPs of P1 and P2 are empty as unstirred fluids at room temperature are provided. The insert vessels of P3 and P6 are made of stainless steel for improved thermal contact.

### Safety features of the robot

Operator collisions with the moving robot elements are dangerous. Therefore, the work area is enclosed using transparent macrolon safety screens. If these screens are opened during a run, processing is immediately paused and an alarm is triggered until the windows are closed again. The correct program execution is controlled by numerous error detectors on the head, the gripper, the platforms, the thermometers and manometers. Malfunctions immediately trigger an alarm via the parallel I/O bits. The system is set to wait until the operator decides how to proceed. A coordinate test of the movable pipettor ensures correct positioning and prevents collisions. One of the pipetting tips activates a test pin at defined coordinates within the work area. If the pin is not hit an x-y failure alarm is triggered. The accuracy of the z-axis is tested via a micro switch operated via the pin. By stepwise upward movement of the tip the system checks whether the micro switch switches back at the expected z test coordinate. The manual activation of an emergency switch by the operator immediately stops the movement of the gantry axes by disconnecting the power supply. During acetone washing and evaporation dedicated ventilation at the S1/S2 and the A1 and B1 platforms minimizes the dangerous concentrations of acetone in the work area.

### Software for system processing and teaching

The PC operating system is Windows 2000 Professional. The programmes for BASY96 system processing and teaching are written in Agilent VEE, a graphical programming language. The program is structured into about 340 functions (subroutines), which are repeatedly called during the process execution. The processing software used is dedicated to the control of the two interlaced high-throughput isolation processes. Programme changes by the user are not supported. A separate teaching programme can be used to define the x, y, z1, z2, z3 coordinates of the fluid vessels and the coordinates of the DWP platforms and the centrifuge.

### Cooling platforms and cooling fluid

Cooling to temperatures below 0°C requires prevention of water condensation and ice generation. For low temperature processing tasks, a special 4°C cooling box and a -20°C compartment are installed on the work area. Each compartment is constructed as a cooling tray. It is isolated using hard foam and provides space for 8 DWPs. The tray structure prevents condensation caused by airflow and allows access of the gripper from the top. The top of the -20°C compartment is covered with an automatically operated lid.

### Shaking platforms

As the centrifuge allows the simultaneous centrifugation of 4 DWPs, the platforms of the work area are likewise designed for the uptake of 4 DWPs. To avoid time-consuming transport and queues by having only one dedicated shaking station for the frequent shaking steps during processing the platforms that require shaking were designed as shaking platforms. Both shaking and locking of the DWPs is performed by pneumatic cylinders. Each An/Bn shaking platform pair has an electronic hardware controller where the pre-selection of shaking frequencies is set. Via the PC program two pre-defined shaking frequencies can be activated by setting or resetting two control bits. When switched on, the shaking runs independently of the PC until switched off. In between the program can attend to other tasks on the work platform. The A1/B1 shaking platforms are additionally equipped with heating foils for speeding up the evaporation of acetone.

### PC and electronic hardware

The PC hardware consists of a midi tower standard IBM compatible personal computer with a Pentium 4 CPU. The 5-axis gantry system (ELBAG Electronics, Weisel, Germany) and the communication with the centrifuge are controlled via the serial 1 port and the serial 2 port, respectively. Two additional 48 bit parallel I/O interface boards (ME 1400B, Meilhaus Electronic, Puchheim, Germany) are connected to the PCI bus. The 96 control bits programmed as outputs or inputs are used for controlling the function of the pipetting head, the gripper, the platforms, the fluid vessels, the S1/S2 station and the pipetting tip washing station. Additional interface boxes between the I/O boards and the work area house general buffer amplifiers, the shaking platform electronics and the 5 V to 24 V output level converters. A ten button operation box allows the manual up- and down-motion of the five gantry axes and is used for teaching the system.

### Molecular biology experiments

Plasmids were introduced and grown up in E-coli SURE cells (Stratagene). Plasmid DNA was quantified by the ODs at 260 and 280 nm by a Pharmacia GeneQuant photometer. The commercial mini columns (Qiagen) as the most directly comparable system were used to isolate the plasmid DNA according to the manufacturer's recommendations. P1–P5 are the solutions described in Neudecker et al., 2000. P3 is cooled to 4°C, P4 and P5 are stirred to keep silica oxide and SDS resuspended. 293 cells (human embryonic kidney cells) were used in the transfection experiments and were kept in standard DMEM medium with 5% FCS. The transfection protocol was as described in ref 14.

## List of abbreviations

SDS, sodium dodecylsulfate; GFP, Green fluorescent protein; DWP, deep well plates; OD, optical density; PC, personal computer, cDNA, complimentary DNA; DNA, desoxyribonucleic acid, RNA, ribonucleic acid; MTP, multititter plate;

## Authors' contributions

SG and VK conceived and designed the study and drafted the manuscript. VK constructed the robots. G.S contributed the transfections and DNA isolation results. All authors read and approved the final manuscript.

## Supplementary Material

Additional File 1 Additional, detailed information on the robot can be obtained in the supplementary file [see Additional file 1]. Click here for file
