# Supplementary material for: High-throughput isolation of ultra-pure plasmid DNA by a robotic system
Source: BMC Biotechnol. 2006 Feb 16;6:9. doi: 10.1186/1472-6750-6-9 (PMC1395312; doi:10.1186/1472-6750-6-9)
Supplement: Additional File 1 — Additional, detailed information on the robot can be obtained in the supplementary file [see Additional file 1]. [file 1472-6750-6-9-S1.pdf]

Supplementary Information

to

High-Throughput Isolation of Ultra-Pure Plasmid DNA by a Robotic System

Volker Kachel, Georg Sindelar and Stefan Grimm

Contents:

Gantry system

Pipetting head

Gripper

Shaking platforms

Cooling for platforms and acetone

Fluid supply vessels

PC-controlled centrifuge

Pipetting station S1/S2

Wash station for pipetting head

Electronic hardware

Teaching and processing software

Safety features of the robot

a. **Error messages and error handling**

b. **Acetone exhaust**

Gantry system

Figure S1 shows the three main axes Xa, Xb, Y and Z1 of the 5-axis gantry system all of which are driven by stepping motors. The various parts of the robot are commercial products (ERO Führungen, Löffingen, Germany, www.ero-fuehrungen.de). The 96-tip pipetting head and the gripper (Fig. S2) are mounted on the Z1 sled. The motor Mx of the X-axis moves the Y-Z1 bridge along the Xa and Xb rails via tooth-belts. The motor Mx drives the tooth-belt of Xa directly and the tooth-belt of Xb via the shaft Sx. The Y drive is also based on a tooth-belt. The Z1 axis is driven by a spindle.

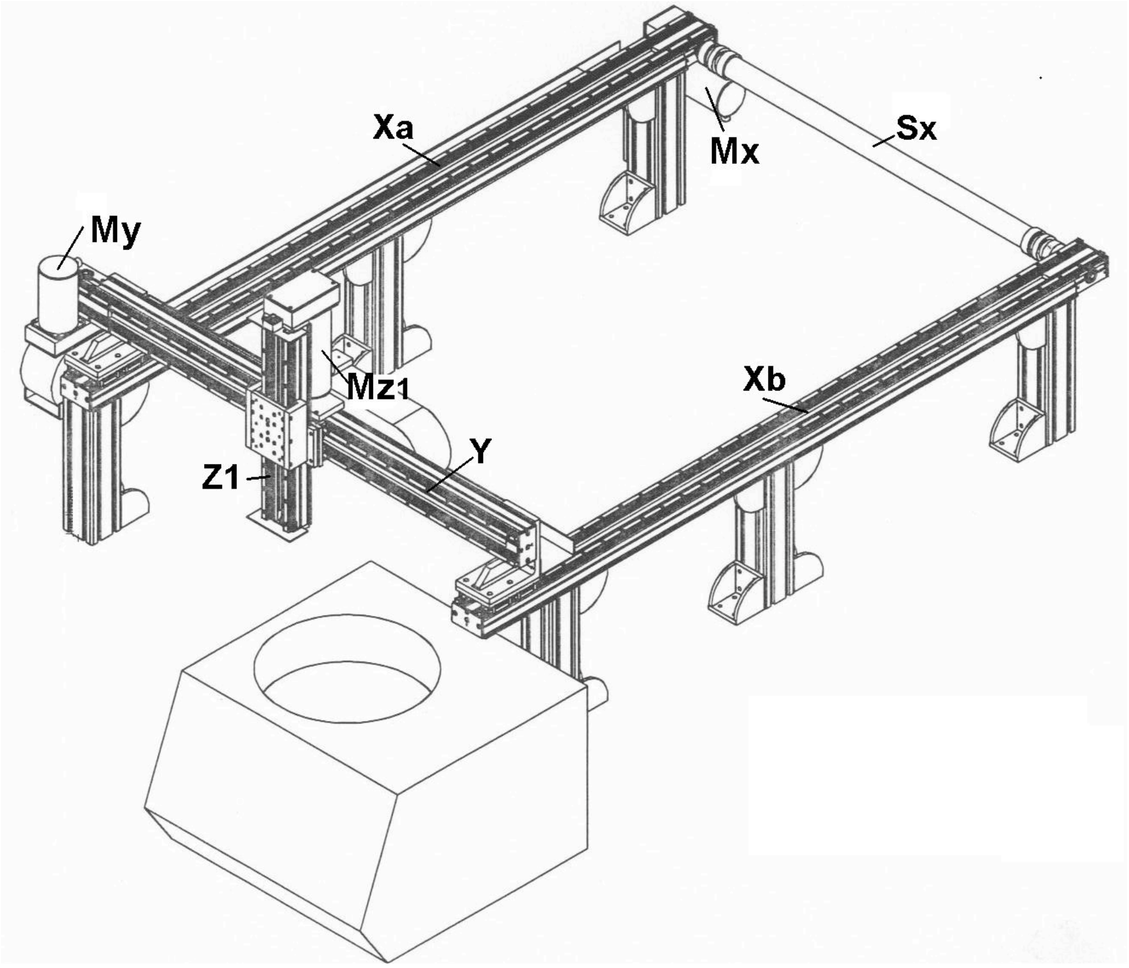

Fig. S1: The three main drive axes Xa, Xb, Y and Z1 of the 5-axis gantry system with the stepping motors Mx, My and Mz1. The shaft Sx interconnects the tooth-belt drives of the axes Xa and Xb.

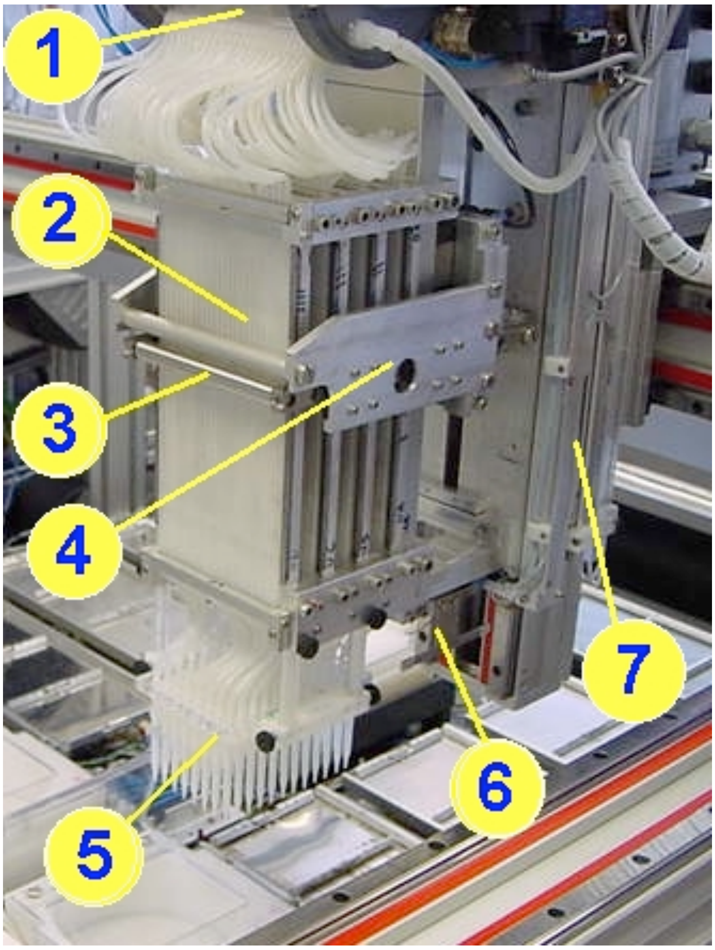

Fig. S2: The 96-tip pipetting head with gripper. (1) common vessel; (2) pipettor tubes; (3) roller; (4) frame of the 4 rollers; (5) pipetting head with 96 tips; (6) hand of the gripper; (7) linear element moving the hand down and up

Pipetting head

The 96-tip pipettor (Fig. S2) comprises 96 parallel tubes arranged in four levels of 24 tubes each[1]. The upper ends of the tubes (2) are connected to a common vessel (1), which is normally under low air pressure. The lower ends are connected to pipetting tips (5). Four rollers (3) guided by the up- and down-moveable frame (4) can be pressed onto the tubes. By moving upwards they operate like the pistons of syringes and suck fluid into the tips. At the respective destinations the rollers are released and the contents of the tips is blown out by the air pressure in the vessel. A second functional principle is also realised by the rollers. They are pressed onto the tubes while the common vessel is under a slight vacuum. For the uptake of fluid the rollers are released for a few milliseconds without up- or down-motion. Thus, they function like a 96-fold multi-valve[1]. The volume taken up in that way by the tips depends on the strength of the vacuum and the time between the release and the re-engagement of the rollers. Dispensing can be achieved in the same way but by using a positive air pressure in the vessel. The second operation principle is much faster compared with the first operation mode but less accurate. It is used in the process for washing the pipetting tips.

Gripper

The gripper is mounted directly behind the movable pipettor. The gripping hand ((6), Fig. S2) is driven by an HGP-16-A pneumatic element (Festo AG, Esslingen, Germany). A micro-switch sensor detects whether a DWP has been taken up. The gripping hand is mounted on the bottom end of a pneumatic linear unit (SLG-12-300-P-A, Festo AG, (7)) which can move

the gripping hand 20cm down from its top position. The exact z base position for taking up a DWP is adjusted using the Z1 sled, with the pneumatic linear unit in top position. From there the linear unit moves the hand 20cm down to the actual pick-up point on the platform.

Shaking platforms

Six shaking platforms are available on the work area of the robot. They are used both as static platforms and as linear shakers for the DWPs (Fig. S3 and S4). The static part mounted on the base plate (2) is shown in red colour. During loading, unloading and static use the shaking cylinder (1), which is activated by the valve (4), holds the shaking platform (11) in the left position that is exactly defined by the delimiter (7,7a). The rotating locks (17) are released (open) and the microswitch (5, 5a) signals “loading coordinates ok”. Positioning of the DWPs is facilitated by the guiding frames (12). For shaking, the piston of the cylinder (1), which is connected to the shaking part by (8), moves the platform (11) periodically forward and backward between the stops of the delimiter (7,7a). The moving part is guided by rolls (10), which are mounted on the frame (9) and move on the shaking rail (6). With the

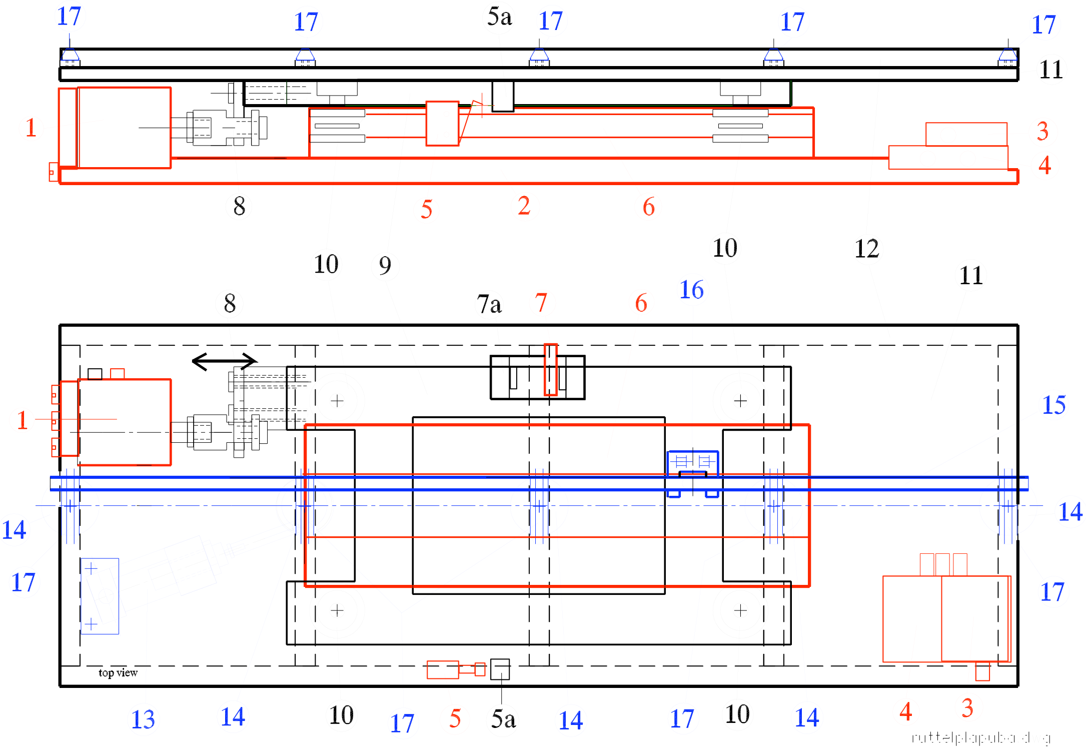

Fig. S3: Mechanic and pneumatic elements of the shaking platforms: red:static base plate:; (1) shaking cylinder; (2) base plate; (3) locking valve; (4) shaking valve; (5) microswitch controlling the basic position; (5a) limit stop of the microswitch; (6) shaking rail; (7) shaking limit stop; **black: moving part** (7a) U-shaped shaking delimiter; (8) shaking drive connection; (9) frame of the shaking rolls; (10) shaking rolls; (11) shaking platform; (12) guiding frame around the DWP positions; **blue: locking system** (13) locking cylinder; (14) disk-shaped locking drives; (15) connecting bar; (16) locking delimiter; (17) rotating locks

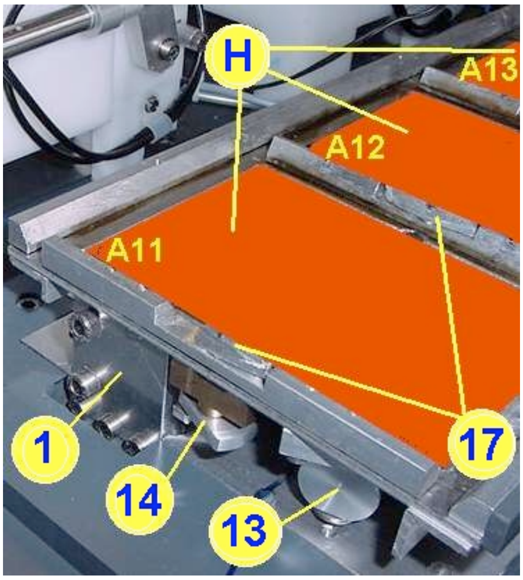

Fig. S4: Shaking platform A1 with integrated heating foils (H). See Fig. S3 for an explanantion of the various items

start of the shaking the locks (17) fix the DWPs in their positions using the locking cylinder (13), which is activated by a valve (3). They are connected by means of the disk-shaped locking drives (14) and the connecting bar (15) whose motion is limited by the locking delimiter (16). At the end of shaking the locks are released. Figure S5 shows the hardware controllers for a pair of An/Bn (n=1,2,3) shaking platforms. They contain variable shaking clocks, clock dividers by factor of 2, 4, 8 and 16, PC controlled switches for two shaking frequencies and drivers to convert the voltage for use in the 24V pneumatic valves. The manually adjustable clocks generate base frequencies, which are reduced by the clock divider to four defined shaking frequencies. The computer can then

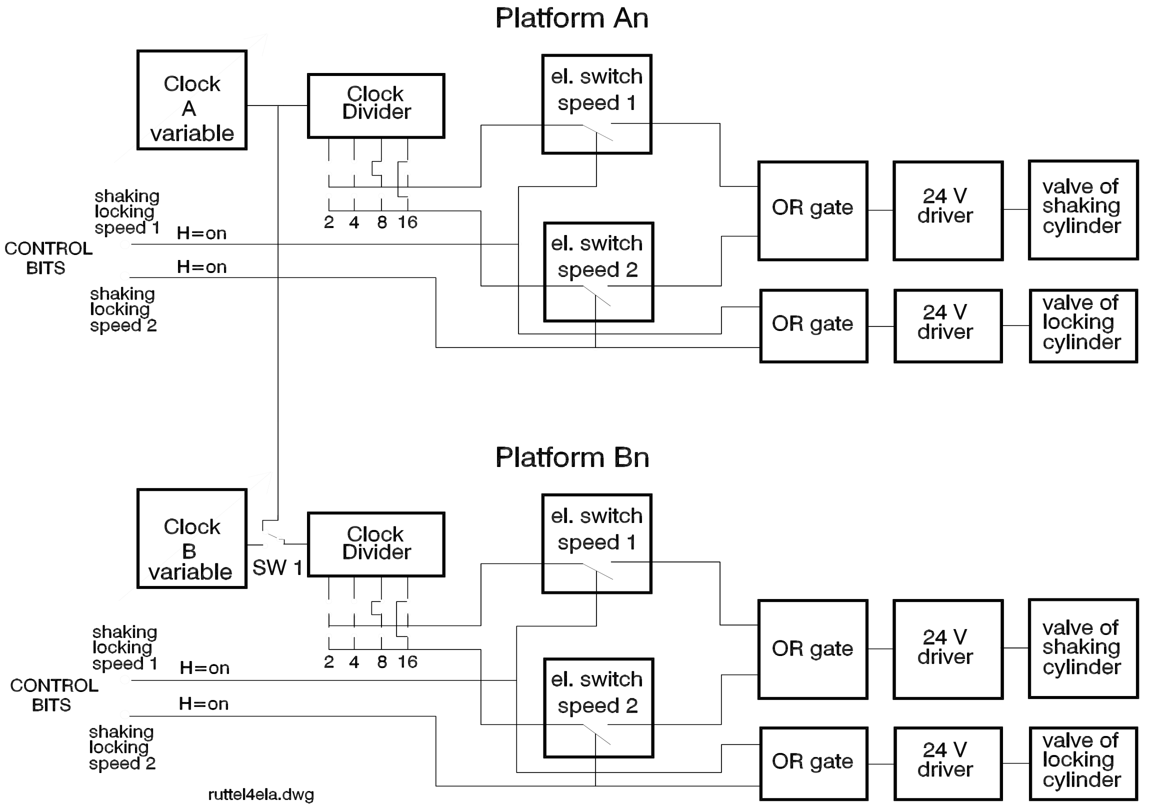

Fig. S5: Block circuit of the shaking hardware of a pair of An/Bn shaking platforms

address two of these shaking frequencies by specific bits. The OR Gates make sure that the actually addressed frequency is used for the movement of the platform and that both bits activate also the locking cylinder. Thus, the PC control bits switch “shaking/locking” with speed 1 or speed 2 while in between the program can attend to other tasks. If the switch SW1 is connected to clock A both platforms An and Bn are synchronised. Since the circuits are laid out for shaking of A and B in opposite directions the vibrations of the robot platform are reduced. Switch SW1 connected to Clock B makes platform B independent of the shaking frequency of platform A.

C. Cooling for platforms and acetone

A –20°C and a 4°C cooling platform are installed in the work area. Both provide four spaces

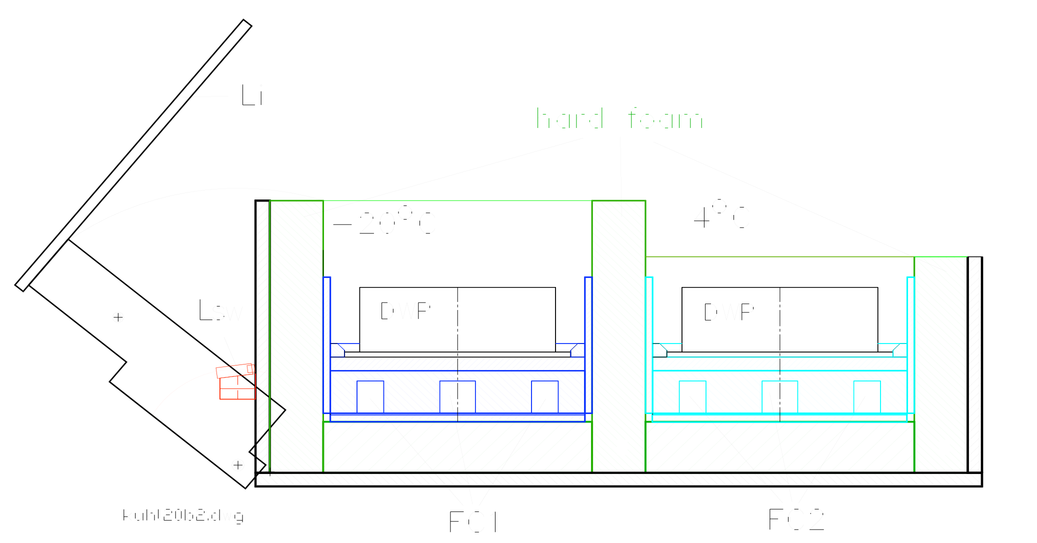

Fig. S6: Cross section through the –20°C and 3°C cooling box; (Li) pneumatically operated lid of the –20°C compartment; (Lsw) Microswitch controlling the lid; (FC1) Cooling fluid of –20°C; (FC2) Cooling fluid of 4°C

each for DWPs from process A or process B. The –20°C compartment is encased and covered by a pneumatically operated lid (Li) to prevent water condensation and ice generation (Fig. S6). Hard foam of 3 cm thickness is used for thermal insulation. The cooling platforms consist of aluminium blocks with internal fluid channels (FC1 and FC2). The –20°C and 4°C cooling fluids are provided by flow coolers located underneath the work area. About 1l of acetone per run is required for washing the plasmid DNAs. In order to reduce vaporisation and to retain vapour generated the acetone is stored in a thermally isolated and cooled box located underneath the S1/S2 pipetting station (Fig. S7). The cooling fluid for the cooling block of the box and the cooled acetone dispensing tray (4) is provided by a –20°C flow cooler via thermally isolated tubes (1, 2, 3). Also, the cooled acetone is pumped through thermally isolated tubes (5, 6) into the thermally isolated dispensing tray.

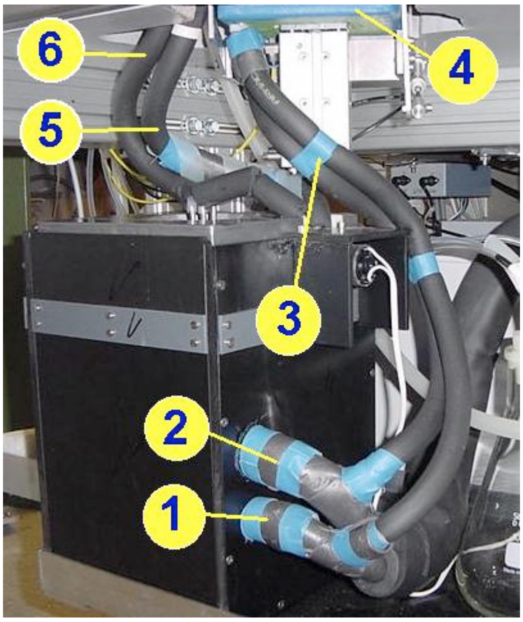

Fig. S7: The cooling box supplying the acetone for washing the plasmid DNAs

Fluid supply vessels

The fluid vessel holder has an opening (1 in Fig. S8) to insert vessels as shown in Fig. S9. The lids (2) are moved by pneumatic cylinders (4). The throttle valves (4a, 4b) are set for smooth opening and closing of the lid. Fixing elements (3a and 3c) are used for an additional cover plate between holder and lid with a rectangular entry opening (not shown) in order to reduce the evaporation of the fluid during opening of the lid. The legs of the vessel holders are held in place on the work plate using angles ((11) in Fig. S10). In between the legs there is space for mounting additional functional elements like electronic stirrers, heaters or coolers (Fig. S10). The electronic stirrer (10) (Variomag compact, H+P Labortechnik, Oberschleissheim, Germany) is used for fluids P4 and P5. The stirrer is permanently running during the process and its speed is manually adjustable (12). The insert vessels are generally polyethylene. Stainless steel vessels are only used for cooled (P3) or heated (P6) fluids. With these vessels the openings of the holders are bottomless and the vessels sit directly on the heaters or coolers for optimal thermal contact. The thermal blocks have the same form as the stirrer. The cooling block of fluid P3 is supplied by a 4°C cooler and the 60°C block of P6 is electrically heated and controlled.

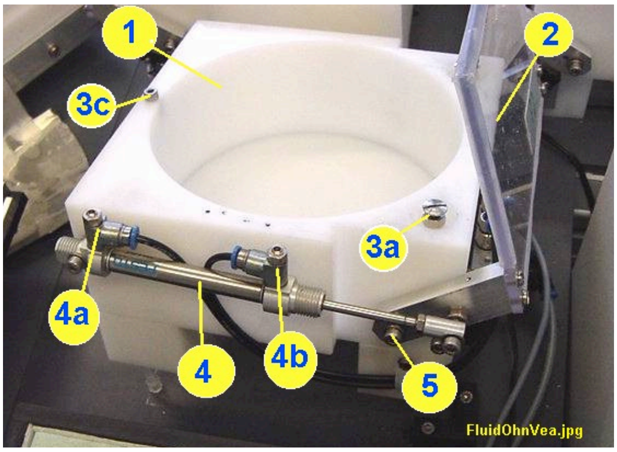

Fig. S8: Fluid vessel holder (1); (2) tiltable lid; (3a, 3c) fixing elements for cover plate (not shown); (4) pneumatic cylinder of the lid; (4a, 4b) throttle valves; (5) bearing of the lid

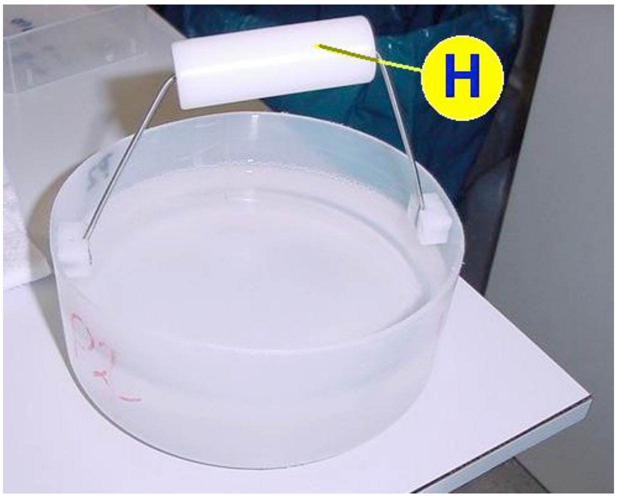

Fig. S9: Insert vessel with removable handle (H)

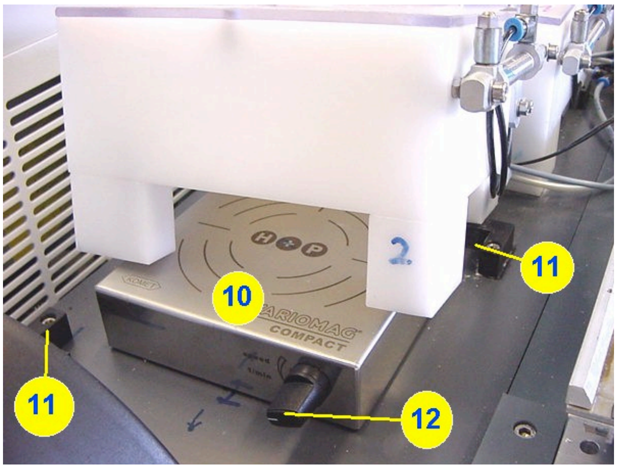

Fig. S10: Electronic stirrer (10) in the space below the opening of a holder. (11) Fixing angles on the working plate, (12) adjustment of stirring speed

PC-controlled centrifuge

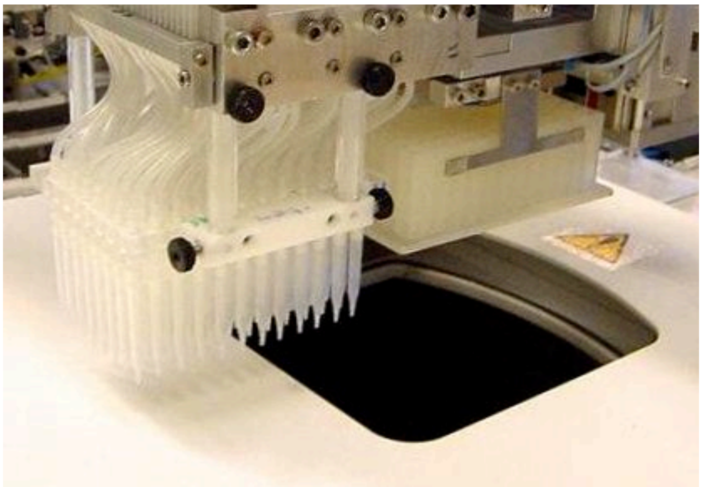

Fig. S11: The gripper holding a DWP waits to load the centrifuge through the loading hatch in its lid.

The PC-controlled centrifuge (SIGMA 4K15 robotic, Sigma, Osterode, Germany) is equipped with a four-position rotor for the centrifugation of DWPs. The positions, numbered 1 to 4, can be precisely positioned under the loading hatch of the lid (Fig. S11). The rotor is turned with low speed to the pre-selected position and fixed using a mechanical pin, which is inserted into the rotor. This means that each of the 4 positions is loaded and unloaded from the same gripper coordinates. A dedicated imbalance procedure during the start of the centrifugation was implemented to deal with possible imbalance stops (the centrifuge was designed by the manufacturer to only stop upon detecting imbalances). Figure S12 explains the start routine of the centrifuge including handling of imbalance stops. In section (A) the commands for centrifugation time and revolutions/min are transmitted to the centrifuge via serial2. From the “until break” object the programme branches into section (B). The programme repeats (B) until the status bits indicate that the rotor is spinning and accelerates. The range between 500 and 2000 rev/min is critical for imbalance. The count object in section (C) defines that section (D) will be repeated four times for the evaluation of the bits reporting imbalance of the centrifuge. Normally, i.e. when no imbalance occurs during the critical acceleration range, section (D) and thus also the start function is exited after the fourth repeat via section (F). In the event of an imbalance, the programme branches from section (D) to section (E) where an alarm is triggered, the gripper is moved away from the lid of the centrifuge and the program is set to wait. The supervisor can now open the centrifuge and take out the DWPs for rebalancing. After repositioning the DWPs and re-closing the lid clicking on “Imbalance ok” continues the program: the centrifuge and the cooling temperature are reset, and the previous centrifugation time and number of rpm is transmitted. The programme execution returns to section (D) and from there to exit section (F). The panel view (Pa) shows the part of the function visible on screen during execution.

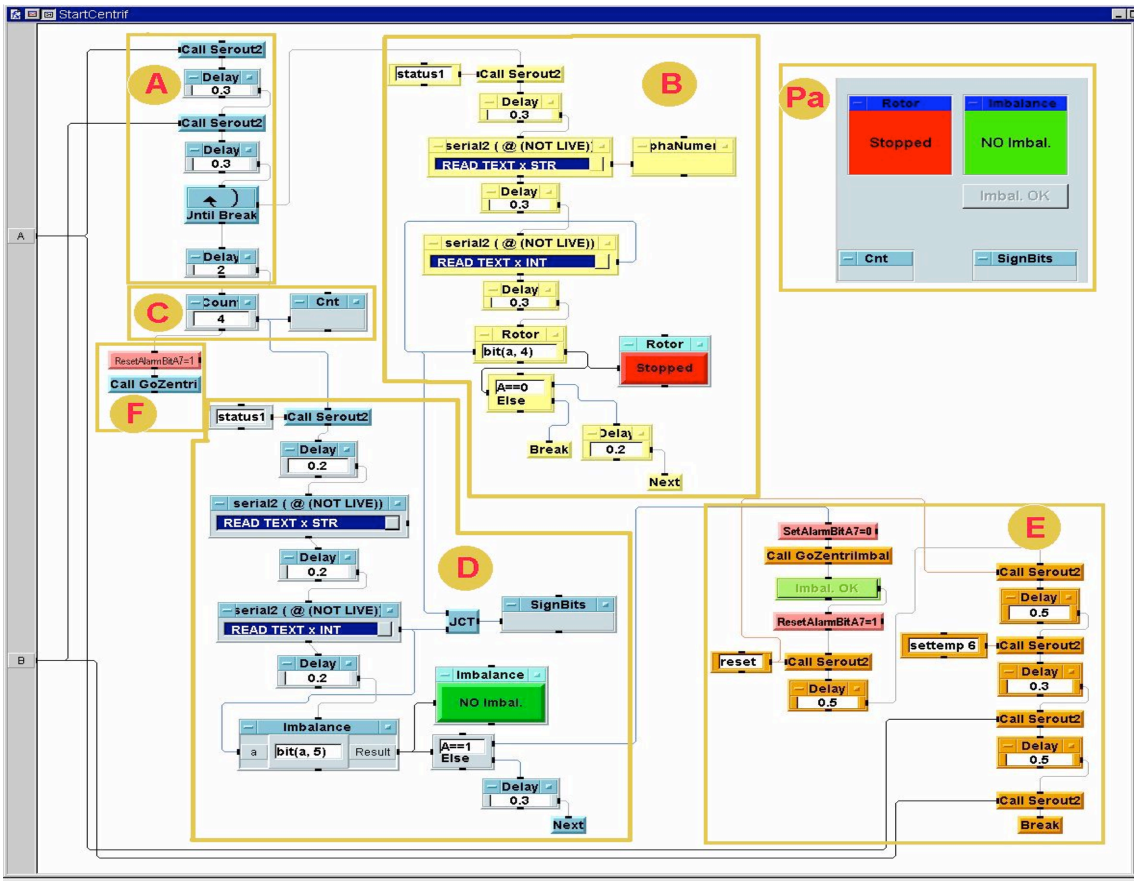

Fig. S12: The VEE program function (subroutine), which starts the centrifuge under imbalance control with error handling (A-D). Restart of the centrifuge after imbalance detection and manual DWP re-adjustment by the system supervisor (E, F). Inputs to the function (ASCII strings) at the left hand side strip: (A) centrifugation time; (B) revolutions/min

Pipetting station S1/S2

All three pipetting heads (S1a, S1b, S2a) of the independent pipetting station S1/S2 (Fig. S13) work according to the "suction-pressure" principle[1] (Fig. S14) in which suction and pressure are generated in a common air tube and relayed to the 96 pipetting tips. This requires that the pipetting tips are selected for uniform outlet openings. Fig. S15 explains how tips are selected from mixed sizes delivered by the manufacturers. Air flows from the pump (P) through the throttle (th) and the tip to be tested which is plugged on the test cone (TC). Depending on the flow resistance of the tip, which is determined by the size of its opening, the digital pressure meter (DM) measures a characteristic pressure value. The device is controlled by measuring a reference tip ("Ref") in certain time intervals. If its pressure value

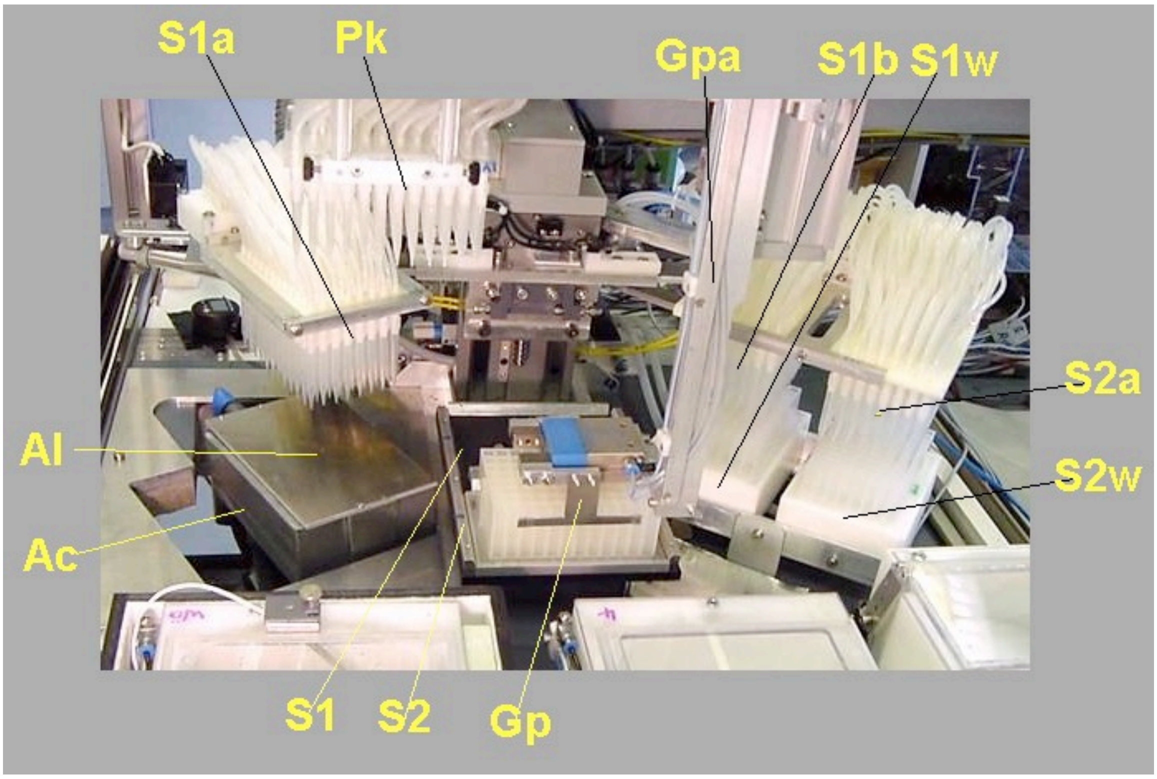

Fig. S13: The separate pipetting station S1/S2. The pipetting heads (S1a), (S1b/S2a) can swing out. The platforms S1 and S2 are then availabe for loading. The gripper (Gp) mounted on the arm (Gpa) loads a DWP to platform S2. The lid (Al) of the cooled acetone vessel (Ac) is closed. The collectors S1w and S2w take up the waste from the pipettors S1b and S2a. The main pipettor (Pk) is moved with the gripper without a functional role in the S1/S2 pipetting station.

has changed the initial reference pressure value has to be restored by adjustment of the throttle. The tested tips are classified into pressure groups, which constitute groups of equal openings. Lower pressures correspond to larger openings and vice versa. The mean opening size of the tips has to be considered for the determination of the pipetting volume when

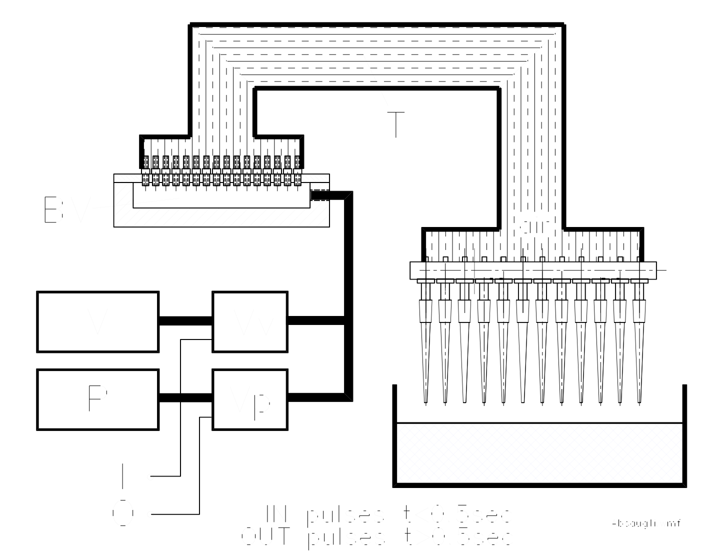

Fig. S14: The valve controlled suction-pressure principle. V vacuum source; P pressure source; Vv vacuum valve; Vp pressure valve; BV branching vessel; T silicon tubes; I input switch signal; O output switch signal;

mounted into one of the pipetting heads. Platform S2, e.g. is used for removing the high volume bacteria supernatant after the first centrifugation in the process.

The 96-tip pipetting heads S1a and the joined heads S1b and S2a (Fig. S13) swing out when a DWP is delivered by the gripper (Gp,Gpa). For the addition and removal of acetone a DWP is placed on platform S1. The cover Al is opened, the cooled acetone (to reduce the acetone vapour pressure) vessel moves up and the pipettor S1a is lowered down until its tips are immersed into the acetone. Using a short suction pulse about 200μl acetone is taken up by the tips. The pipetting system is moved up, S1 is swung over the DWP, moved down until the tips enter the top of the wells and the acetone is blown out into the wells by a smooth pressure pulse. For acetone withdrawal the DWP is also placed on platform S1, pipettor S1b is swung over the DWP, moved down until the tips are immersed into the acetone supernatant and the supernatant is taken up by a short suction pulse. S1b is moved up, swung out over the waste S1w and the content of the pipetting tips is blown out. The number of suctions, the depth of immersion and the strength and duration of the suction pulses are determined experimentally. The complete acetone washing process (Fig. S16) is performed in one cycle that is run twice: Addition of 400μl acetone to the DWPs, transport to

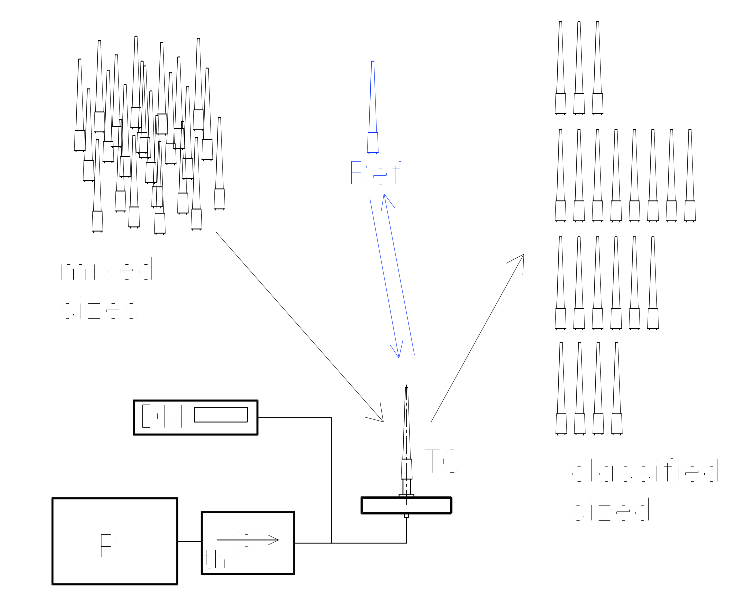

Fig. S15: Selection of pipetting tips of uniform opening sizes: (P) pressure source, (th) throttle; (DM) digital pressure measurement; (TC) testing conus; (Ref) reference tip

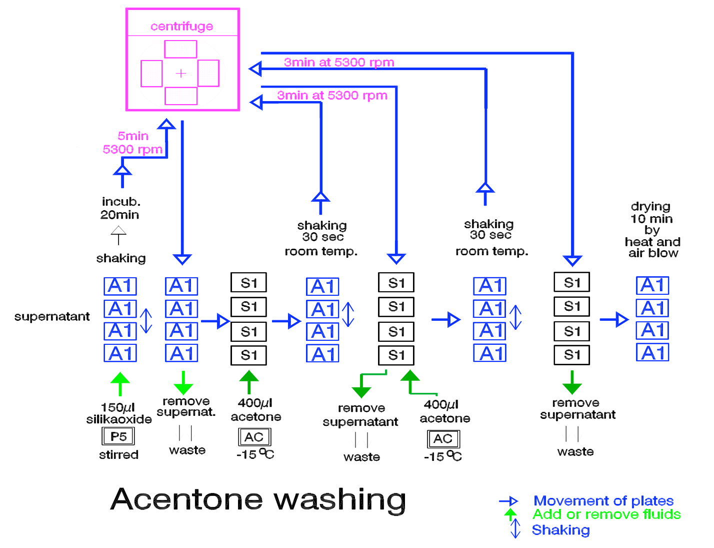

Fig. S16: Schematic diagram of the twofold acetone washing process

A3/B3 platform, shaking, transport to centrifuge, centrifugation, transport to S1, removal of acetone supernatant and addition of 400µl acetone.

Wash station for pipetting head

Each pipetting step of the main pipetting head is followed by washing in the tip washing station (Fig. S17) located at the centre of the work area. The block with the 96 washing tubes (2) is located inside a waste fluid-collecting tray (3). It is designed for efficient internal and external washing of the tips with minimum consumption of washing fluid. Only when the tips to be cleaned are immersed into the washing tubes is the flow of the clean water (blue) switched on by a pulsating membrane pump. The upwards flowing fluid washes the outside of the tips. Simultaneously, water is sucked into the interior, the tips are moved up and positioned over the gaps between the washing tubes where the water is blown out. This water (green) is then collected in (3) and flows out through the outlet (4) to the waste vessel. Any number of such washing cycles can be programmed.

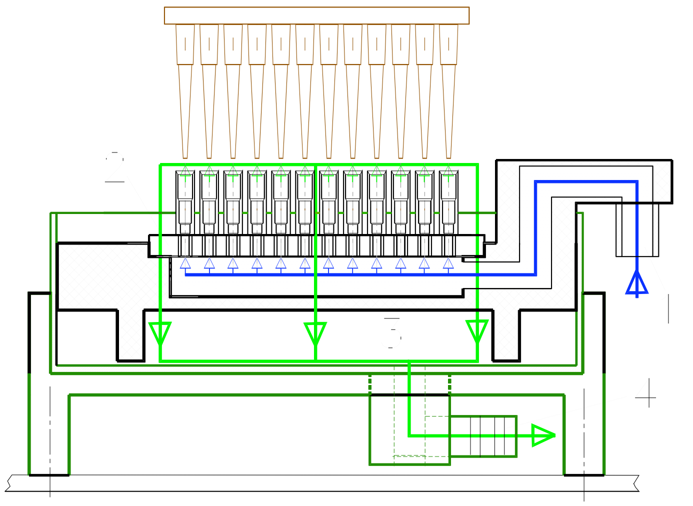

Fig. S17: The pipette tip washer. (1) inlet of fresh fluid; (2) plate with 96 washing tubes; (3) waste collecting tub; (4) waste outlet

Electronic hardware

Figure S18 shows a summary outline of the system hardware. The PC is a standard Pentium IV system (1.5Ghz clock, 40Gb HD, CD) with two additional parallel I/O cards comprising 48 I/O lines each (Meilhaus Electronik, ME1400B). The gantry x-y-z-motion, the pipetting motor and the z-axis of the S1/S2 system are controlled via the serial1 port. The centrifuge is connected to the serial2 port. The other functional commands such as shaking of the platforms, opening and closing of lids and air- or fluid-valves, error messages on mechanical functions, pressures and temperatures are transferred via the interfaces of the parallel I/O system. “Manual Move” of the axes is used for system teaching and the Emergency STOP switch immediately stops the motors of the 5 axes.

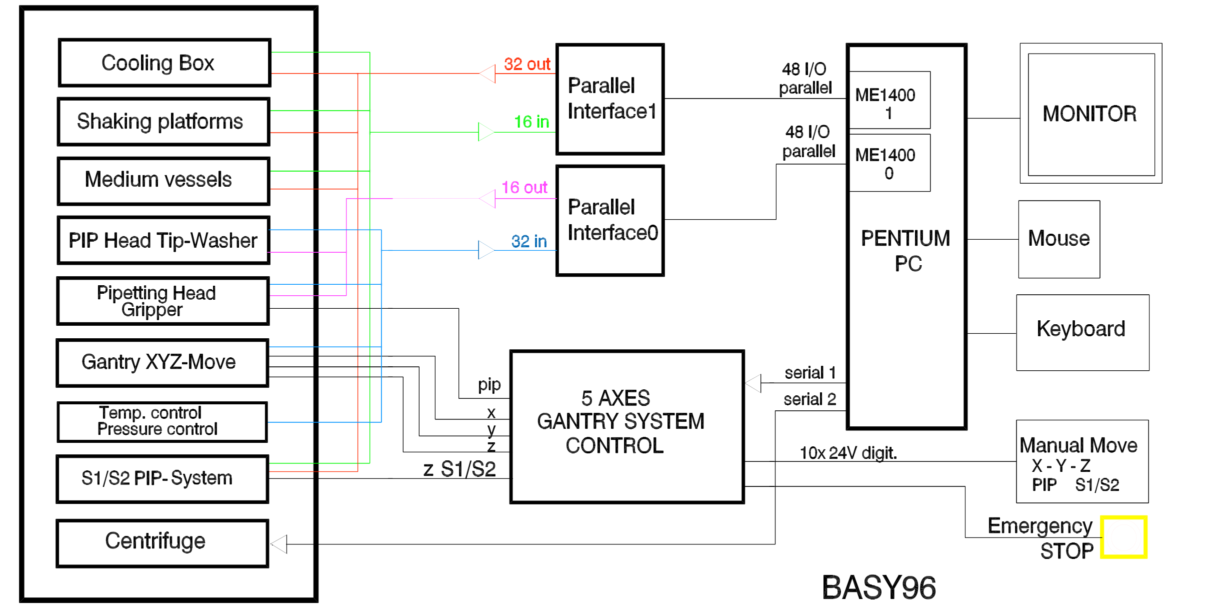

Fig. S18: Summary outline of the BASY96 hardware

Teaching and processing software

The software is written in Agilent (HP) VEE, a suitable graphical programming language. The coordinates for pipetting, uptake and setting down of DWPs are determined via a teaching programme. The system is initialised and the pipetting head or the gripper is manually moved to the correct positions on the work area. The coordinates are shown on the screen for programming the main processing software. The main software is optimised for an interlaced processing of two 384 samples in four DWPs. Figure S19 shows a typical screen shot during a run. Field I indicates the progress of the execution. Each process is divided into 50

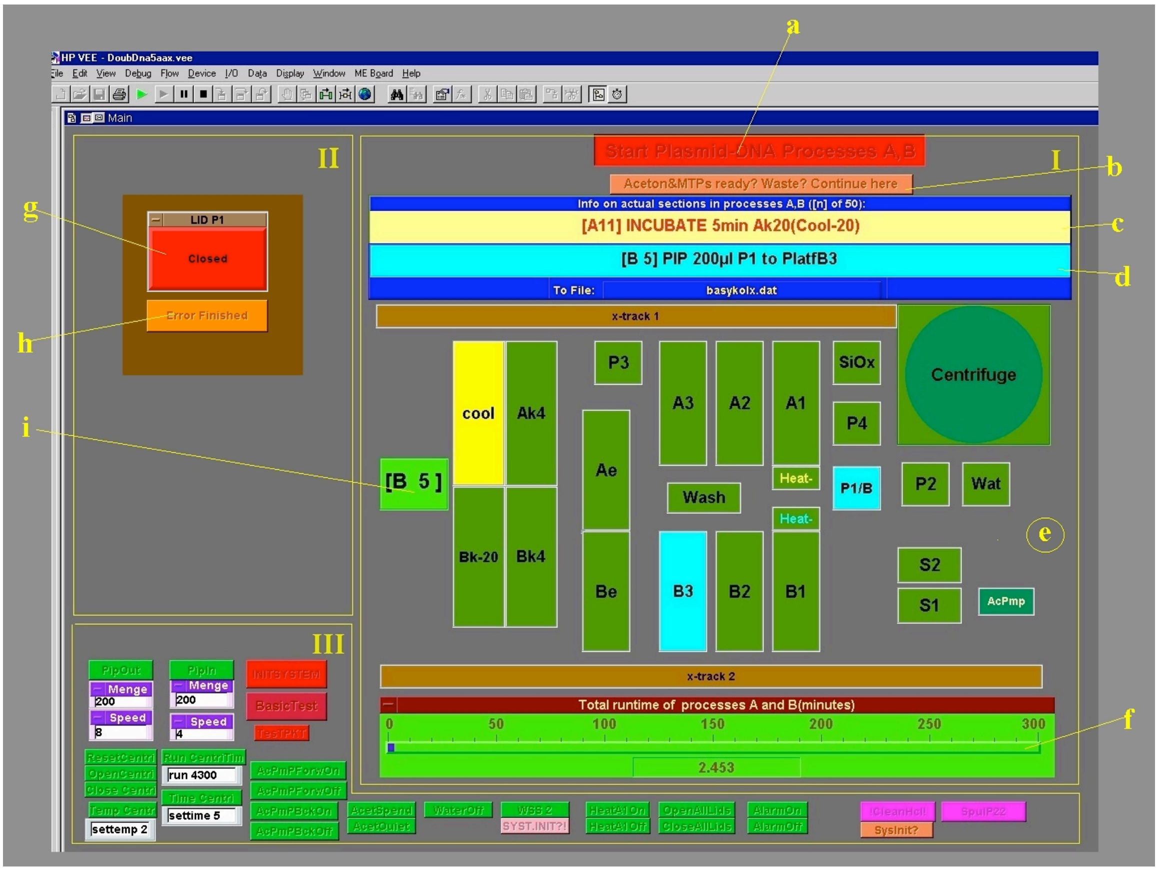

Fig. S19: Typical screen shot during the execution of the two interlaced processes A and B. Field I indicates the progress of the processes. Field II shows errors and general messages. Field III allows manual calling of functions (disabled during run of the main processes).

segments: [An] and [Bn]. The indicator lines (c) for process A and (d) for process B show the actual state of the execution. In the present example process A is in the segment "[A11] INCUBATE 5min Ak20(Cool-20)" and process B is in segment "[B5] PIP 200μl P1 to Platform B3". [B5] on (i) indicates that segment B5 is currently being processed. On the graphical representation of the work area (e) the colour yellow, which represents process A, marks the platform Ak20 (cool). The process B activities are indicated in turquoise on platform B3 and fluid vessel P1. Item (f) shows the overall timescale of the ongoing processes. Field II is only used during the execution time of messages. The message (g) warns that the lid of vessel P1 remained closed and processing is paused. After opening the lid, the user clicks button (h) to continue processing. Correct execution of the segments and error messages are recorded using a real time stamp in the protocol file indicated in the bottom line of (d) (basykolx.dat). Field III offers a collection of functions for manual execution, e.g. testing when the main processes are not executed.

Safety features of the robot

a. Error messages and error handling

During the execution of the processing programme safety-relevant functions are supervised and the result shown in the error-info-panel on the screen (Fig. S20). When errors are detected, the error control field changes the colour, an error specifying message is shown, an acoustic alarm is triggered and an error message with time stamp is written into the protocol file. Also, the programme execution changes into the error-wait state. The supervisor may now try to clear the fault. He can then continue processing by clicking the confirmation field in the error message field. In the event of a serious fault the program has to be stopped completely. Fig. S21 shows the situation when the gripper misses a DWP when accessing a platform.

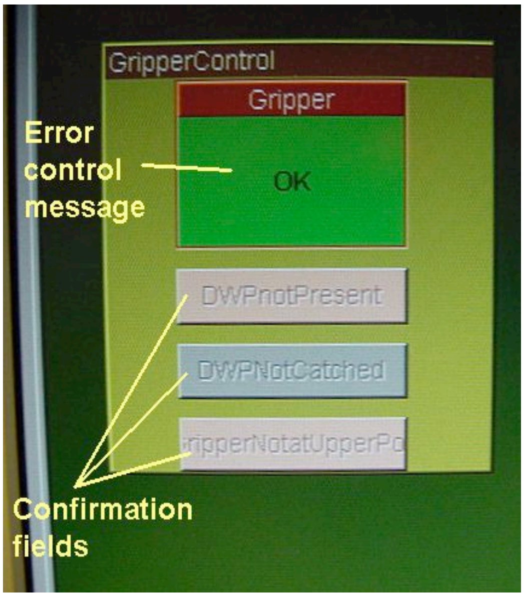

Fig. S20: Error-info-panel on screen with an error control message and confirmation fields for continuation of the programme. In this instance no error has occurred.

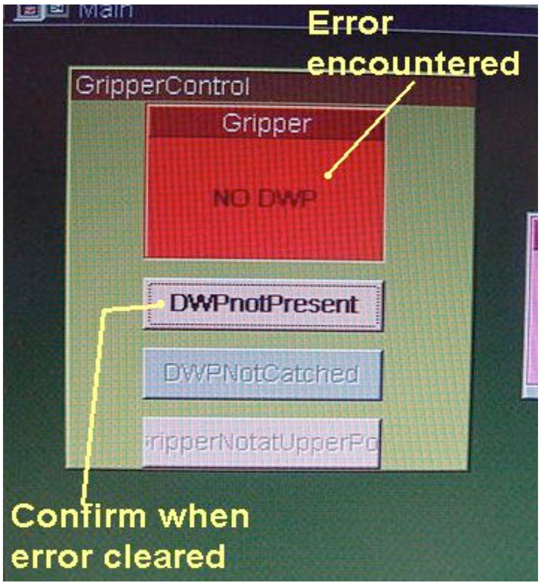

Fig. S21: Error-info-panel in the event of an error. The process is stalled. When the error has been cleared the process may be continued by clicking the confirmation field

List of errors supervised on the working platform:

SECURITY DOORS ....OPEN

WATER OF PIPETTING TIP CLEANING ....EMPTY

PRESSURE 7BAR (PNEUMATICS) ....OUT OF RANGE

PRESSURE 0.9BAR (HEAD) ....OUT OF RANGE

PRESSURE 0.02 BAR (HEAD) ....OUT OF RANGE

SUCTION –0.2 BAR (HEAD) ....OUT OF RANGE

PRESSURE 0.2 BAR ON S1/S2 ....OUT OF RANGE

TEMPERATURE –20° (COOLING BOX A20/B20)............OUT OF RANGE

TEMPERATURE 4° (COOLING BOX A4/B4) ....OUT OF RANGE

GRIPPER...........NOT AT UPPER POSITION

GRIPPER ....DOES NOT FIND A DWP ON ACTUAL POSITION

GRIPPER ....HAS NOT CATCHED THE DWP FROM ACTUAL POSITION

ARM 1 OF S1/S2 ....NOT AT INSIDE TILTED POSITION

ARM 2 OF S1/S2 ....NOT AT INSIDE TILTED POSITION

S1/S2 ....NOT AT UPPER POSITION

LID OF P1 ....NOT OPEN

LID OF P2 ....NOT OPEN

LID OF P3 ....NOT OPEN

LID OF P4 ....NOT OPEN

LID OF P5 ....NOT OPEN

LID OF P6 ....NOT OPEN

LID OF COOLING BOX A20/B20 ....NOT OPEN

SHAKE-STOP-POSITION OF PLATFORM A1 .....NOT OK

SHAKE-STOP-POSITION OF PLATFORM A2 .....NOT OK

SHAKE-STOP-POSITION OF PLATFORM A3 .....NOT OK

SHAKE-STOP-POSITION OF PLATFORM B1 .....NOT OK

SHAKE-STOP-POSITION OF PLATFORM B2 .....NOT OK

SHAKE-STOP-POSITION OF PLATFORM B3 .....NOT OK

b. Acetone exhaust

A dedicated exhaust is connected to the laboratory ventilation system to remove the acetone vapour generated during the washing process of the plasmid DNA and during the subsequent drying of the silica pellets (Fig. S22). A trap in the ventilation head directs the main suction to the inlet (2) when the acetone wash on platform S1 is in progress. During the drying process when the DWPs on the platforms A1 and B1 are heated up and drying air is successively blown into the wells by the pipetting head, the inlet (3) is activated. It is connected to the suction tube (4), which absorbs the acetone vapour generated.

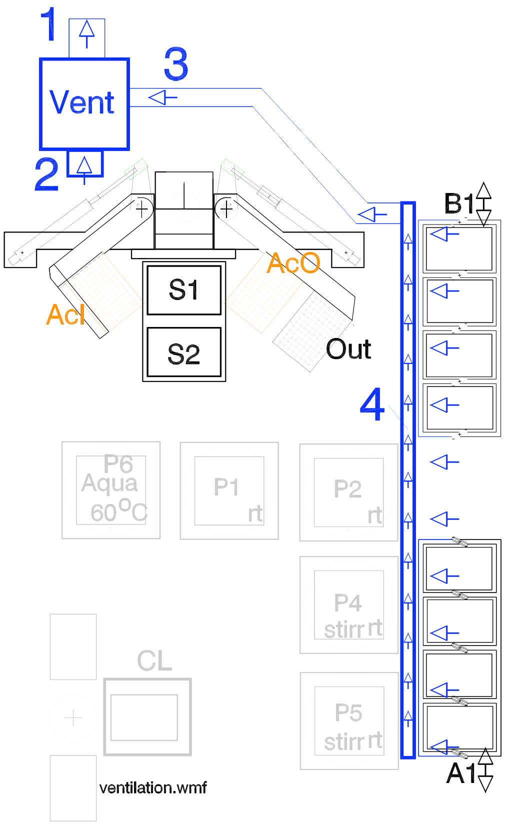

Fig. S22: Exhaust of acetone vapour; (1) connection to ventilation; (2) inlet from the S1 area; (3) inlet from platforms A1, B1; (4) collection tube for the area of the platforms A1 and B1

1. Grimm, S. & Kachel, V. Robotic high-throughput assay for isolating apoptosis-inducing genes. *Biotechniques* **32**, 670-2, 674-7. (2002).
